# Fatty Acid Content and Composition of the Yakutian Horses and Their Main Food Source: Living in Extreme Winter Conditions

**DOI:** 10.3390/biom10020315

**Published:** 2020-02-17

**Authors:** Klim A. Petrov, Lyubov V. Dudareva, Vasiliy V. Nokhsorov, Kirill N. Stoyanov, Olesia N. Makhutova

**Affiliations:** 1 Institute for Biological Problems of Cryolithozone of Siberian Branch of the Russian Academy of Sciences, 41 Lenina av., Yakutsk 677000, Russia; kap_75@bk.ru; 2 Siberian Institute of Plant Physiology and Biochemistry, Siberian Branch of Russian Academy of Sciences, 132 Lermontova str., Irkutsk 664033, Russia; laser@sifibr.irk.ru; 3 North-Eastern Federal University, 48 Kulakovskogo str., Yakutsk 677000, Russia; nohvasyavas@mail.ru; 4 Siberian Federal University, 79 Svobodny pr., Krasnoyarsk 660041, Russia; ikirill97@gmail.com; 5 Institute of Biophysics of Federal Research Center “Krasnoyarsk Science Center” of Siberian Branch of Russian Academy of Sciences, Akademgorodok, Krasnoyarsk 660036, Russia

**Keywords:** essential polyunsaturated fatty acids, linoleic acid, alpha-linolenic acid, food quality, muscle tissue, subcutaneous adipose tissue, liver, green cryo-fodder

## Abstract

For the first time, seasonal changes in the content of total lipids (TLs) and phospholipids (PLs) were studied in fodder plants growing in Central Yakutia—a perennial cereal, smooth brome (*Bromopsis inermis* L.), and an annual cereal, common oat (*Avena sativa* L.). Both species have concentrated TLs and PLs in autumn under cold hardening. In addition, a significant increase in the content of fatty acids (FAs) of *B. inermis* was observed during the autumn decrease in temperature. The Yakutian horses, which fed on cereals enriched with nutrients preserved by natural cold (green cryo-fodder), accumulated significant amounts of 18:2n-6 and 18:3n-3, the total content of which in cereals was 75% of the total FA content. We found differences in the distribution of these two FAs in different tissues of the horses. Thus, liver was rich in 18:2n-6, while muscle and adipose tissues accumulated mainly 18:3n-3. Such a distribution may indicate different roles of these FAs in the metabolism of the horses. According to FA content, meat of the Yakutian horses is a valuable dietary product.

## 1. Introduction

The Republic of Sakha (Yakutia), located between 105°32′–162°55′ E and 55°29′–76°46′ N, occupies the territory of 3103.2 thousand km^2^ and lies completely in the permafrost zone in Russia. During the short growing season, plants are exposed to high activity of solar radiation, moisture deficiency, and short-term frosts on the soil surface in early summer and autumn. Native plant species growing in such extreme conditions adapt to going through all the stages of ontogenesis in a shorter time period [1,2]. At different stages of ontogenesis, the ability of plants to adapt to cold hardening is not the same: the closer the plant is to the reproductive phase, the lower its ability to adapt to cold [3]. More than 2000 species of higher vascular plants grow in the permafrost zone of Yakutia, which is an unusual phenomenon [4]. Some of them play an important role as a food source for herbivores.

A specific feature of the seasonal growth and development of the bulk of vegetation in the permafrost zone is that its intensive growth occurs in the first half of summer. However, at this time, northern meadow plant communities are often covered with floodwaters and are also subjected to grazing and haying. After traumatic regeneration, the plants do not have time to go through the full cycle of growth and development, produce fully developed seeds, and stay in a green frozen state under the snow cover in winter (green cryo-fodder). The basis of cool-season grass is cereals, which preserve up to 80% of herbage under snow, as well as sedge, cotton grass, and some horsetails [5,6]. The wintergreen parts of the above families of fodder plants retain higher contents of proteins, carbohydrates, and fats for the winter compared to warm-season grass [7,8].

Green cryo-fodder is the basis of nutrition for many animals, including the Yakutian horses. This breed is considered a direct successor and descendant of the horses brought from the Baikal region in the 13th–15th centuries AD [9,10,11]. The origin of the horses was confirmed by molecular genetic methods [12,13,14,15].

The Yakutian horse demonstrates unique adaptation to long-term low-temperature stress, which has been achieved in a short evolutionary period [16]. The reasons for such good adaptation have not been fully studied. Feeding on green cryo-fodder may help animals survive in extremely cold winters [16]. 

The aim of the present work was to study lipid accumulation in a perennial cereal (*Bromopsis inermis* L.) and an annual cereal (*Avena sativa* L.) cultivated at different temperatures. Additionally, we aimed to study the content and composition of fatty acids (FAs) in liver, muscle, and subcutaneous adipose tissues of Yakutian horses, which have *B. inermis* and *A. sativa* as part of their staple diet.

## 2. Materials and Methods

The annual cereal common oat (*Avena sativa* L., Nyurbinsky type) was sown on 31 May 2014 (control) and on 15 July 2014 (treatment). The perennial cereal smooth brome (*Bromopsis inermis* L., Ammachaan type) was mowed after spring growth to allow the aftergrowth in the middle of summer (15 July 2014)—the treatment, and it was compared with the unmown plants—the control. The experiments were carried out in field plots in the conditions of Central Yakutia (near Yakutsk, 62° N, 130° E). Samples of the control and treatment plants were taken, depending on the phases of development and hardening, 4–5 times during the growing season.

For the analysis of total lipids of the common oat, the control samples were taken 4 times from July 7 to July 25, 2014; and the treatment samples were taken 4 times from July 25 to September 30, 2014. For the analysis of total lipids of the smooth brome, the control samples were collected 4 times from June 6 to July 25, 2014; and the treatment samples were collected 5 times from July 25 to September 30, 2014. To analyze phospholipids of the common oat and the smooth brome, the controls were sampled on July 25 and June 16, respectively; and the treatments of both plants were sampled on October 3, 2014. To analyze FA composition of the smooth brome, samples of the control were collected on July 7, 2013 and those of the treatment on September 25, 2013. The FA composition of the common oat (the control and the treatment) was reported in a study by Petrov et al. (2016) [17].

Sampling took place in the first half of the day in three biological replicates. Samples were fixed with liquid nitrogen immediately after their collection, in situ, and transported in Dewar vessels to the laboratory.

The samples of liver, muscle and subcutaneous adipose tissues were collected in November 2017 and 2018, from female and male Yakutian horses, most of which were less than 1 year old. Four female horses were seven, eight, and eighteen months old and five years old; and two male horses were eight months old, and one male horse was seven months old. The horses were feeding on green cryo-fodder during three months before sampling. Muscle and adipose tissues were carved from the costal part of the animals. The samples were collected from horses inhabiting Oymyakonsky, Verhoyansky, Megino-Kangalassky, Churapchinsky, Olekminsky, and Suntarsky districts of Yakutia.

Large pieces of horse tissues (200–300 g) were immediately frozen and kept at −20 °С at the slaughter site. Then, in approximately 2 weeks, frozen tissues were transported to the laboratory. In the laboratory, samples were taken from the frozen horse tissues, placed into vials with chloroform and methanol (2:1, *v*/*v*), and kept at −20 °С for further analysis.

### 2.1. Conditions of Keeping and Feeding the Horses

The absolute annual temperature difference in the breeding area of Yakutian horses exceeds 100 °C (the maximum summer and winter temperatures are +38 °C and −70 °C, respectively). The frost period lasts 7–8 months a year. In such conditions, the herds of Yakutian horses (12–15 individuals) are kept in the open. The horses are mainly fed on cereal grains and sedge frozen by natural cold. Horse breeders feed only weakened, emaciated individuals and mares. The weight of a breeding stallion reaches 430–520 kg and the weight of a mare 415–480 kg. In our study, we mainly used tissue samples from 6–8-month-old horses taken from local horse breeders. At this age, horse’s tissues have a high nutritional value. Mass slaughter was conducted in November, when horses reached an average of 120–150 kg of live weight, having accumulated the largest amount of fat. For most of their lives, horses fed exclusively on warm-season grass and green cryo-fodder. The biochemical content and the composition of blood of Yakutian horses are described in detail in the literature [18].

### 2.2. Analyses of Lipids and FAs of Plants

A weighed portion of plant material (0.5 g) was ground to obtain homogeneous mass [19]. Then, it was supplemented with 10 mL of the chloroform: methanol mixture (2:1, *v*/*v*), and ionol was added as antioxidant (0.00125 g per 100 mL of the chloroform: methanol mixture). The resulting mixture was thoroughly stirred and left for 30 min until the lipids completely diffused into the solvent. The solution was transferred quantitatively to a separatory funnel through a paper skim filter (9 cm in diameter, Khimreaktivkomplekt); the mortar was washed three times using the same solvent mixture. For better delamination, water was added.

For the analysis of total lipids, the chloroform fraction was separated. Chloroform was removed from the lipid extract using an RVO-64 rotary vacuum evaporator (Czech Republic). Nonadecanoic acid (C19:0) was used to control the extractability of lipids (%), with its known amount added at the stage of homogenization. Methyl ethers of fatty acids (FAMEs) were obtained using the method [20]. For additional purification of FAMEs, TLC method was used in a chromatographic chamber with benzene as the mobile phase (*R*_f_ = 0.71–0.73) on glass plates with silica gel. The FAME zone was removed from the plate with a spatula and eluted from the silica gel with (*n*)-hexane. The FAME analysis was performed on the gas chromatograph Agilent-6890N coupled to an Agilent-5973 quadrupole mass spectrometric detector (Agilent Technologies, USA). The ionization method used was electron impact; the ionization energy was 70 eV. The analysis was carried out in the recording mode of the total ion current. An HP-INNOWAX capillary column (30 m × 250 μm × 0.50 μm) with a polyethylene glycol stationary phase was used to separate the FAME mixture. The carrier gas was helium, the rate of gas flow was 1 mL/min.

The temperature of injection port was 250 °C, the temperature of the ion source was 230°C and that of a quadrupole was 150 °C. Scanning was performed in the range of 41–450 atomic mass units. The volume of the injected sample was one μL, the flow divider was 5:1. The separation of the FAME mixture was carried out in isothermal mode at 200 °C. The duration of the chromatographic course was 60 min. For identification of FAs, the NIST 08 and WILEY7 mass spectral libraries were used. The relative content of FAs was determined by the method of internal normalization, i.e., as weight percent (wt.%) of their total content in the sample, taking into account the response factor of FAs. The absolute content of total lipids and FAMEs was determined by weighing them on GR-120 electronic scale (A&N Company Ltd., Japan) after drying the samples to constant weight.

Separation of PL fractions into individual lipids was carried out by thin layer chromatography (TLC) on Sorbfil PTLC-AF-V-UV chromatographic plates (10 × 10 cm, Russia). For the detection and identification of phospholipids in plant material, specific reagents were used: molybdenum blue for phosphorus-containing components [21], Dragendorf reagent prepared according to the method described by Wagner et al. [22] for choline-containing lipids, and a 0.2% solution of ninhydrin in acetone for amino-containing lipids [23].

Quantitative determination of phospholipid content was carried out according to the Vaskovsky method [21]. The polar lipids were separated using a two-dimensional system: the mobile phase in the first direction—chloroform—methanol—benzene—28% NH_4_OH, 65:30:10:6, and the mobile phase in the second direction—chloroform—methanol—acetic acid—acetone—benzene—water, 70:30:4:5:10:1. To determine the phosphorus content in phospholipids separated by TLC, the silica gel from the zones containing separated phospholipids was transferred with a micro spatula into the tubes; 0.05 mL of 72% perchloric acid was added to each and heated at 180–200 °С for 15–20 min, placing the tubes in a heated aluminum block so that the top of the tube served as an air cooler for perchloric acid vapors. After cooling, 0.45 mL of working reagent was added to the tubes: a mixture of 5.5 mL of universal molybdate reagent, 26 mL of 1N sulfuric acid, and 68.5 mL of distilled water. The reagent was used for one week. The mixture in the tube was thoroughly mixed using a shaker. The tubes were placed in boiling water bath for 15 min and then cooled; the absorbance value was measured at 815 nm. An aliquot of the solvent containing the lipid extract was taken as a blank sample [21]. The air temperature in the experimental area was recorded using a DS 1922L iButton thermograph (Dallas Semiconductor, USA).

### 2.3. Analyses of FAs in Animal Tissues

The samples (0.2–1.3 g) of intercostal muscle, subcutaneous adipose tissue, and liver were homogenized, and lipids were extracted with chloroform and methanol (2:1, *v*/*v*). Dry lipids were then supplemented with 1 mL of sodium methylate solution in methanol (8 g/L). The mixture was heated for 15 min at 90 °C. The tubes were cooled, supplemented with 1.3 mL of methanol: H_2_SO_4_ (97:3, *v*/*v*), and methylated for 10 min at 90 °C. The FAMEs were extracted from the mixture with 2 mL hexane and washed three times with 5 mL of saturated NaCl solution. The hexane extract containing FAMEs was dried by passing it through a layer of anhydrous Na_2_SO_4_, and then the layer of anhydrous Na_2_SO_4_ was washed with 6 mL of hexane. Hexane was evaporated on a rotary vacuum evaporator. FAMEs were resuspended in 0.1 to 0.3 mL hexane prior to chromatographic analysis.

Analysis of fatty acid methyl esters was conducted using a gas chromatograph with a mass spectrometric detector (Model 7000 QQQ, Agilent Technologies, USA), which was equipped with a 30 m capillary HP-FFAP column with the internal diameter of 0.25 mm. The conditions of the analysis were as follows: the velocity of the helium carrier gas was 1.2 mL/min; the temperature of the injection port was 250 °C; the temperature of the heater was programmed from 120 to 180 °C at a rate of 5 °C/min for 10 min isothermally, then to 220 °C with a rate of 3 °C/min for 5 min isothermally, and then to 230 °C at a rate of 10 °C/min for 20 min isothermally; the temperature of the chromatography/mass interface was 270 °C; the temperature of the ion source was 230 °C and that of the quadrupole was 180 °C; the ionization energy of the detector was 70 eV; and scanning was performed in the range of 45–500 atomic units with a rate of 0.5 sec/scan [24]. The data were analyzed and counted by the MassHunter Software (Agilent Technologies). The peaks of fatty acid methyl esters were identified by the mass spectra obtained. The content of fatty acids in the biomass was quantified based on the peak value of the internal standard, nonadecanoic acid (Sigma-Aldrich, USA), a certain amount of which was supplemented to the samples before the extraction of lipids.

### 2.4. Desaturase and Elongase Activity Indices

Desaturase and elongase activity indices were calculated using the product/precursor ratio of the percentages of individual FAs according to the following notation: 16:1n-7/16:0 = Δ9-desaturase, 18:1n-9/18:0 = Δ9-desaturase, 20:4n-6/20:3n-6 and 20:5n-3/20:4n-3 = Δ5-desaturase and 18:0/16:0 = elongase [25]. Additionally, we measured a conversion efficiency of 18:2n-6 to 20:4n-6 (20:4n-6/18:2n-6) and a conversion efficiency of 18:3n-3 to 20:5n-3 (20:5n-3/18:3n-3).

### 2.5. Statistical Analysis

The tables and figures show the averages of three to six biological replicates and their standard errors. Statistical processing of experimental data was carried out using the statistical analysis package in Microsoft Office Excel 2010 and STATISTICA-9 software (Stat Soft Inc., USA). The normality of the distribution of the data obtained was checked using the Kolmogorov–Smirnov one-sample test for normality D_K-S_.

## 3. Results

The contents of total lipids in oat leaves of both the control and the treatment gradually increased as they grew and developed (Table 1). With the decrease in the average daily air temperature from 9 to 1 and −3 °С (periods of the first and second hardening phases), the content of total lipids in oat leaves increased by a factor of 1.2 compared with the control plants of the same stage of development (*t*-test Student’s = 3.34) (Table 1).

In the summertime (June–July), the perennial smooth brome grown without mowing demonstrated lower absolute content of total lipids at all stages of development, i.e., below 60 mg/g dry weight, compared to the aftergrass (Table 2).

Cool-season cereals growing after mowing, which were hardened by low positive temperatures, i.e., when the average daily air temperature reached 1 °C, showed the amount of total lipids 2.4 times higher (Student’s *t*-test = 14.93) compared to the control plants in the same stage of development (Table 2).

**Figure 1:**
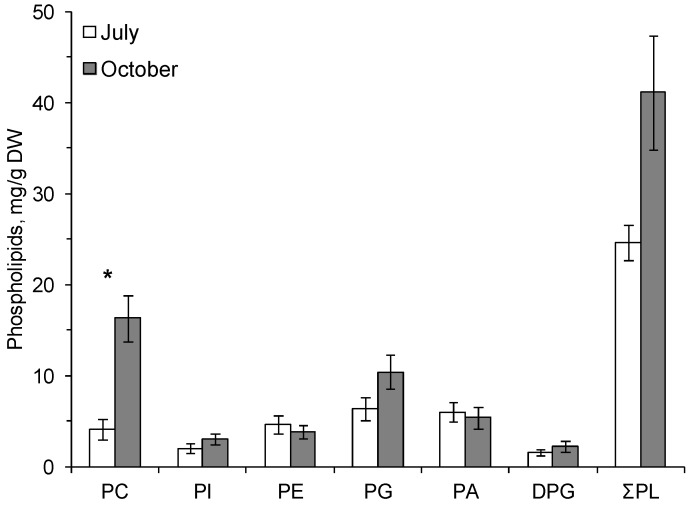
The contents (mg/g dry weight, standard error) of total phospholipids (ƩPL), phosphatidylcholine—PC, phosphatidylinositol—PI, phosphatidylethanolamine—PE, phosphatidylglycerol—PG, phosphatidic acid—PA and diphosphatidylglycerol—DPG in the leaves of *Avena sativa* on 25.07.2014 (July) and 3.10.2014 (October). *—Significant differences according to Student’s *t*-test.

**Figure 2:**
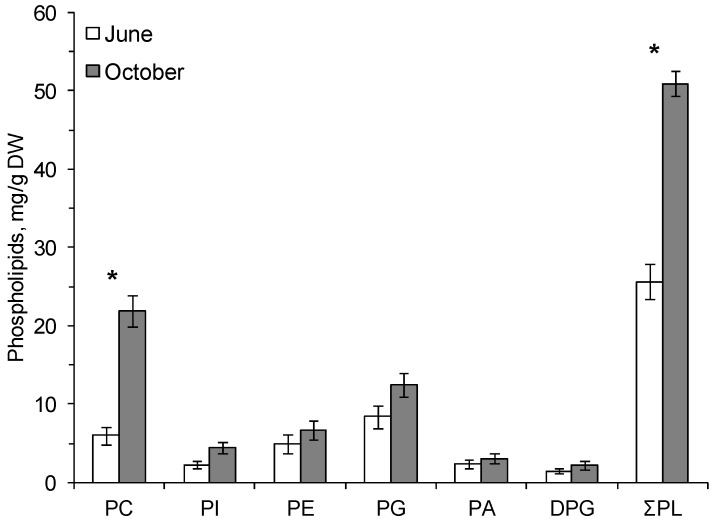
The contents (mg/g dry weight, standard error) of total phospholipids (ƩPL), phosphatidylcholine—PC, phosphatidylinositol—PI, phosphatidylethanolamine—PE, phosphatidylglycerol—PG, phosphatidic acid—PA and diphosphatidylglycerol—DPG in the leaves of *Bromopsis inermis* on 16.06.2014 (June) and 3.10.2014 (October). *—Significant differences according to Student’s *t*-test.

The following phospholipids (PLs) were found in the cereal plants: phosphatidylcholine (PC), phosphatidylinositol (PI), phosphatidylethanolamine (PE), phosphatidylglycerol (PG), phosphatidic acid (PA), and diphosphatidylglycerol (DPG). The dominant PLs were PC and PG (Figure 1 and Figure 2).

In autumn, at the onset of low positive temperatures, the amount of PC increased in common oats by a factor of 4 and in the smooth brome by a factor of 3.7 compared with the content of these phospholipids in summer (Figure 1 and Figure 2). The content of membrane PLs in the leaves of the smooth brome hardened by low positive temperatures significantly increased compared to summer values (Figure 2).

Sixteen fatty acids were identified in all samples of the smooth brome. The quantitatively and qualitatively prominent FAs are shown in Table 3. Among FAs, 18:3n-3, 16:0 and 18:2n-6 dominated, their total content reaching 85–90%. The total content of FAs in the leaves of the brome in the autumn period was significantly (1.8 times) higher than in the summer period (Table 3).

The content of polyunsaturated fatty acids (PUFAs) in the smooth brome leaves significantly increased, while the content of total saturated fatty acids (SFAs) did not change with the decrease in air temperatures (Table 3). The content of 16:0, 16:1 isomers, 18:2n-6, and 18:3n-3 in the leaves of brome in autumn was significantly higher than in summer (Table 3).

The percentage of SFAs in brome leaves was lower in September compared with July. The decrease in SFAs was due to a decrease in the percentage of 16:0, 20:0, and 22:0 (Table 3). The percentage of PUFAs did not change with the decrease in air temperatures while the percentage of 18:2n-6 significantly increased (Table 3).

**Figure 3:**
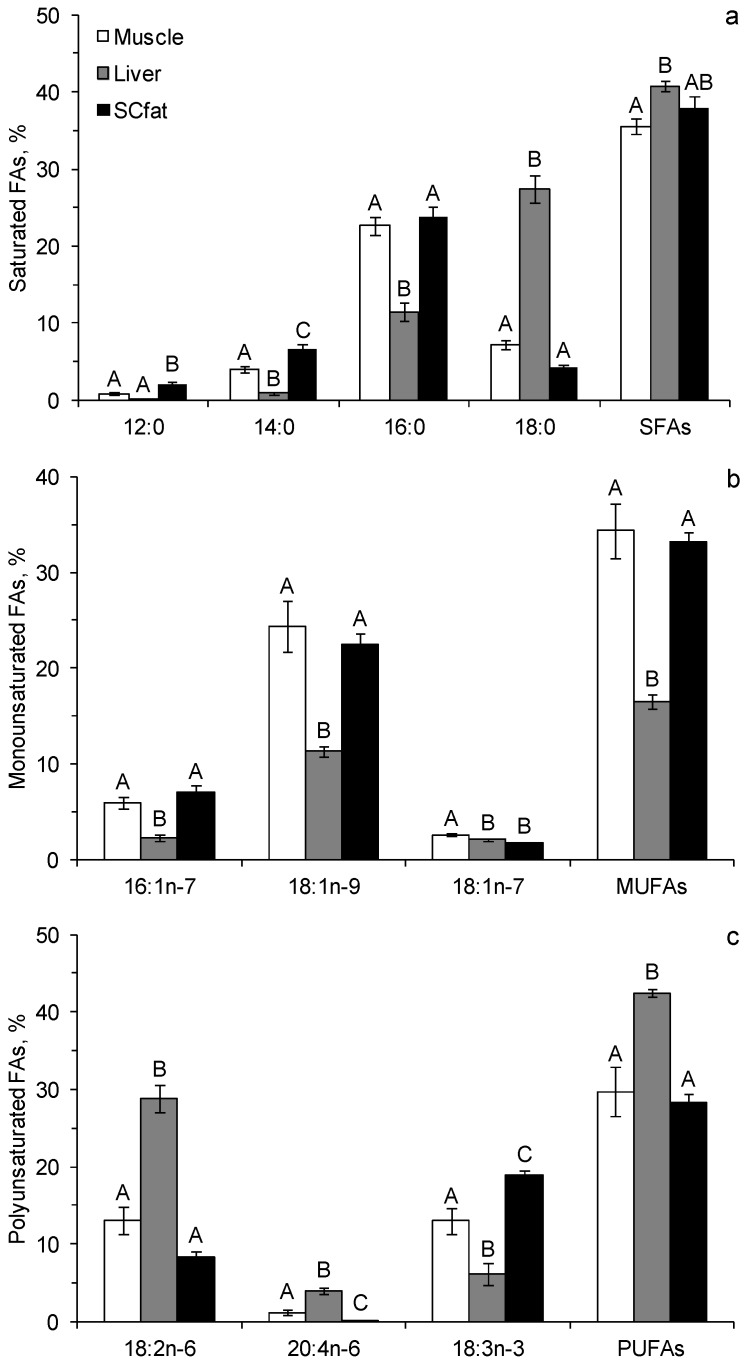
Contents of the prominent saturated fatty acids (**a**), monounsaturated fatty acids (**b**) and polyunsaturated fatty acids (**c**) (% of total FAs, standard error) in liver, muscle and subcutaneous adipose tissues of the Yakutian horses. Means for the same FAs labeled with the same letters are not significantly different at *p* < 0.05 after Tukey’s HSD *post hoc* test.

Fifty-three FAs were identified in the samples of liver, muscle and subcutaneous adipose tissues of the Yakutian horses. The percentages of important and quantitatively significant FAs are shown in Figure 3. The percentage of SFAs in the liver of the animals was significantly higher than in the muscle and adipose tissues (Figure 3a). Among the SFAs in the liver, 18:0 dominated. Its percentage was about 4 and 6 times higher than that in the muscle and adipose tissues, respectively. Shorter-chain SFAs, such as 14:0 and 16:0, dominated in the muscle and adipose tissues, and their percentages were significantly higher than in the liver (Figure 3a). The percentages of monounsaturated FAs (MUFAs) in the muscle and adipose tissues of the horses were twice higher than in the liver (Figure 3b). Among MUFAs, 18:1n-9 dominated in all types of tissues. Nevertheless, its percentage in the liver was significantly lower than in the other tissues (Figure 3b). The percentage of PUFAs was significantly higher in the animal liver than in the muscle and adipose tissues (Figure 3c). Among PUFAs in the liver, omega-6 PUFA, namely 18:2n-6, dominated. Its percentage was more than twice higher than in the muscle and adipose tissues. In contrast, the muscle and adipose tissues were dominated by omega-3 PUFA, namely 18:3n-3. Its percentage was more than twice as high as the percentage of this FA in the liver (Figure 3c). In total, 70% of all FAs in the muscle and adipose tissues were represented by 18:1n-9, 16:0, 18:3n-3, and 18:2n-6 and in the liver by 18:2n-6, 18:0, 16:0, and 18:1n-9 (Figure 3). No trans-FAs were found in the FA tissue of the horses, and the percentage of branched FAs was less than 1% of the total FAs. The percentages of many FAs were similar in the muscle and adipose tissues of the horses. In adipose tissues, however, the percentages of 18:3n-3 and short-chain SFA (12:0 and 14:0) were significantly higher than in muscles, but almost all long-chain PUFAs, including physiologically important arachidonic (ARA, 20:4n-6), eicosapentaenoic (EPA, 20:5n-3) and docosahexaenoic (DHA, 22:6n-3) acids, were absent (Figure 3). The percentages of EPA and DHA in the liver and muscle tissues were insignificant and ranged from 0.1% to 0.3% of the total FAs.

The contents of physiologically important EPA and DHA in the muscle tissue and liver of the horses were similar (Table 4). The n-6/n-3 ratio in the muscle tissue was about 7 times lower than in the liver, but did not differ significantly from that in the adipose tissue. The total content of FAs in the adipose tissue was 30 times higher than that in the muscle tissue and in the liver (Table 4).

The desaturase and elongase activity, estimated by an indirect method (by product/precursor ratio), were significantly different between the adipose tissue and the liver (Table 5).

The conversion efficiencies of 16:0 and 18:0 to 16:1n-7 and 18:1n-9, respectively, were higher in the adipose tissue and the conversion efficiencies of 16:0 to 18:0, 20:4n-3 to 20:5n-3, 18:3n-3 to 20:5n-3, and 18:2n-6 to 20:4n-6 were higher in the liver (Table 5). The conversion efficiency of 18:2n-6 to 20:4n-6 was higher than the conversion efficiency of 18:3n-3 to 20:5n-3 both in the liver and in the adipose tissue (Student’s *t*-test = 2.75 and *t* = 2.66, respectively). The conversion efficiency of 20:4n-3 to 20:5n-3 and 20:3n-6 to 20:4n-6 in the liver did not differ significantly (Student’s *t*-test = 1.09).

## 4. Discussion

Lower ambient temperatures significantly affect the ‘liquidity’ of plant membranes, reducing their fluidity. This leads to the increased expression of the genes responsible for FA desaturation [26]. The increased fraction of unsaturated FAs in plants with the temperature decrease stabilizes membrane fluidity and restores physiological activities of the associated enzyme and electron transport systems, photosynthesis in particular [27,28,29]. Affected by low temperatures, the genes that encode the synthesis of desaturases involved in the formation of 18:2n-6 and 18:3n-3 are activated in the plants [26,30]. The increase in total lipids, phospholipids, and total FAs that we detected in the cereals showed that these substances along with sugars, proteins, antioxidants, and carotenoids [1,31] are involved in the cold adaptation of cool-season plants in the cryolithozone of Central Yakutia. In the same way as *B. inermis* studied in our work, other herbaceous plants (*Avena sativa, Elytrigia repens, Equisetum variegatum*, and *Equisetum scirpoides*) accumulated significantly more FAs in their vegetative organs during the period of winter cold adaptation than in summer [17,32,33,34].

During cold adaptation of plants, the contents of phospholipids and polyunsaturated fatty acids increase in their tissues [35,36,37]. However, in contrast to most published data, we found a significant increase in the content of phosphatidylcholine but not in the other phospholipids. This finding probably shows the key role of phosphatidylcholine in temperature adaptation of the cereals.

Along with *Bromopsis inermis* and *Avena sativa*, the ability to cryopreserve green mass was found in many other plants in Central Yakutia, for example, cereals—*Arctophila fulva*, *Deschampsia borealis*, *Puccinellia jacutica*, *Poa alpigena*, hydrophytic sedges—*Carex rhynchophysa*, *C. atherodes*, *C. vesicata*, *C. enervis*, and most cotton grasses—*Eriophorum scheuchzeri*, *E. vaginatum*, *E. russeolumsubsp. leiocarpum*, *E. angustifolium* [6]. In the pre-winter period of fat accumulation, herbivores actively consume cool-season and winter-green parts of these fodder plants with the high contents of nutrients [2].

The main consumer of green cryo-fodder in plant ecosystems of cryolithozone in Central and North-Eastern Yakutia is the Yakutian horse. In autumn, from August to the beginning of October, the horses feed on green cryo-fodder. In favorable years, the accumulation of fat on green cryo-fodder by the Yakutian horses lasts up to mid-November [38,39].

The tissues of the Yakutian horses and their fodder were rich in two PUFAs, namely 18:3n-3 and 18:2n-6. These FAs are essential for the majority of animals [40,41,42]. Vertebrates can synthesize physiologically important long-chain PUFAs—20:5n-3, 22:6n-3, and 20:4n-6—from their dietary precursors 18:3n-3 and 18:2n-6, respectively, but the rate of synthesis is generally ineffective [40,43]. Suagee et al. found that mesenteric adipose tissue in horses had a high lipogenic capacity followed by subcutaneous adipose tissue and then liver [44]. Very low percentages of 20:5n-3 and 22:6n-3 (the average value = 0.4% of total FAs) as well as a low 20:5n-3/18:3n-3 ratio in the tissues probably indicated a low conversion efficiency of omega-3 PUFAs in the Yakutian horses. The conversion of omega-6 PUFAs seemed to be more efficient than conversion of omega-3 PUFAs, at least in the liver. The literature data and our results suggest that dietary sources of 20:5n-3, 22:6n-3 and 20:4n-6 were absent from the diet of the Yakutian horses [17]. Thus, we suppose that these long-chain PUFAs were synthesized in horses’ tissues. According to our data, the efficiency of elongation of 16:0 to 18:0 was significantly higher in the liver while the conversion efficiency of SFAs to MUFAs was higher in the subcutaneous adipose tissue. Similar trends in the conversion efficiency of SFAs to MUFAs and elongation of 16:0 to 18:0 in liver and subcutaneous adipose tissue were reported by Adolph et al. for Warmblood horses [25]. 

Different contents of C18, C20, and C22 PUFAs in the tissues of the horses may indicate different functions of these PUFAs. Unlike Warmblood horses, the subcutaneous adipose tissue of the Yakutian horses was rich in PUFAs, especially in the omega-3 family [25]. Mordovskaya et al. and Slobodchikova et al. also noted enrichment of the Yakutian horses’ adipose tissue with 18:3n-3 [45,46]. High percentages of 18:3n-3 in the subcutaneous fat of the Yakutian horses may increase fluidity (liquidity) of adipose tissue during the winter period of extremely low temperatures. This may be a reason for high mitochondrial activity in adipocytes, which increases energy production at low ambient temperatures. The beneficial effects of omega-3 PUFAs on the thermogenic function of adipocytes have recently been demonstrated [47,48,49]. Thus, we suppose that 18:3n-3 served as an energy-related component in the horses. By contrast, omega-6 PUFAs, namely 18:2n-6 and 20:4n-6, which were accumulated in liver and muscle tissue, likely served as important structural components or precursors of lipid mediators. Similar results were reported in a study of the FA composition of different lipid classes in Iberian horses [50]. In the muscle tissue, 18:2n-6 and 20:4n-6 accumulated in polar lipids, apparently performing a building function, and 18:3n-3, on the contrary, accumulated in neutral lipids, performing an energy function [50].

The PUFA content in the horse muscle and adipose tissues varies greatly depending on the diet, breed, and age of the animals [51,52,53]. For example, the content of 18:2n-6 in horse muscles varied from 12% to 32%, and the content of 18:3n-3 varied from 0.43% to 23.9% [53]. Along with individual fatty acids, the total contents of SFAs, MUFAs and PUFAs can also vary greatly in the horse muscle tissue: 34.2–47.8%, 16.4–50.2% and 15.6–46%, respectively [53]. The muscle tissue of the horses we studied contained equal proportions of SFAs, MUFAs, and PUFAs, which corresponded to the minimum SFA values and the average MUFA and PUFA values available in the literature. Similar to the Yakutian horses, horses that were fed on the native grass pasture had the same percentages of SFAs, MUFAs, and PUFAs, as well as high percentages of 18:3n-3, in subcutaneous adipose tissue [54]. The total FA content of the subcutaneous adipose tissue of the Yakutian horse corresponded to the high values known for horses, varying between 457 and 904 mg/g wet weight [53,54]. Obviously, nutrition has a significant effect on the variability of FA percentages in horses. Horses eating fresh plant food, but living in a mild climate, had similar contents and distribution of FAs, including 18:2n-6 and 18:3n-3, to those in the Yakutian horses. High contents of lipids and FAs such as 18:3n-3 and 18:2n-6 in green cryo-fodder probably help the Yakutian horses successfully survive the extreme temperatures of Central Yakutia. However, the results obtained only indirectly indicate this and do not allow us to clarify the subject. 

In contrast to many farm animals, horses are able to efficiently assimilate PUFAs from plant food owing to the structure of their gastrointestinal tract, activity of microorganisms, and the presence of specific pancreatic lipases related with protein 2 (PLRP2) [53,55]. Thus, horse meat is considered as a useful dietary product, i.e., a source of essential PUFAs, namely 18:2n-6 and 18:3n-3 [56,57,58], and can be potentially enriched with long-chain omega-3 PUFAs, 20:5n-3 and 22:6n-3. However, our data suggest that meat, subcutaneous fat, and liver of the Yakutian horses are not rich in 20:5n-3 and 22:6n-3 if their food does not contain these PUFAs. This may indicate limitation in PUFA synthesis in the horses’ tissues. The contents of 20:5n-3 and 22:6n-3 (% and mg/g wet weight) in the liver of the Yakutian horses and other horse breeds were significantly lower than in the liver of cows, pigs, and chickens [59]. The contents of 20:5n-3 and 22:6n-3 (mg/g wet weight) in the meat of horses were the same as in beef and higher than in pork [53,60]. In general, because of the high content of 18:3n-3 and the optimal ratio of n-6 to n-3 PUFAs, the Yakutian horse meat is a more valuable and health food product compared to beef, pork, and chicken, which is consistent with the data of many authors [45,60,61,62]. 

## 5. Conclusions

The cereal plants studied (*B. inermis* and *A. sativa*) accumulate lipids, phosphatidylcholine and fatty acids, in particular, during the period of natural cold hardening in extremely cold climates of the permafrost zone. Cereals enriched with nutrients are the basis for the Yakutian horse feeding during pre-winter fat accumulation. The muscle and adipose tissues and liver of the horses contained high percentages of 18:2n-6 and 18:3n-3, which were abundant in the cereals studied in this work. A likely reason for the diverse distribution of these FAs in tissues is that these FAs perform different functions in the animals. 18:2n-6 is probably used as a precursor in the synthesis of physiologically valuable 20:4n-6, while 18:3n-3 mainly performs an energy-related function. Such a high content of 18:2n-6 and 18:3n-3 in the tissues of horses of the Yakutian breed apparently helps animals successfully survive the extreme temperatures of Central Yakutia, although more research is needed. Additionally, the Yakutian horse meat has proved to be a valuable dietary product due to its low n-6/n-3 ratio.

## Figures and Tables

**Table 1:** Contents of total lipids (TL, mg/g dry weight ± standard error) in the leaves of the annual cereal *Avena sativa* sown on May 31 and July 15, 2014 at different stages of development and growing at different temperatures.

Date	t, °С *		Stages of Development	TL, mg/g DW
	min	Average		
Control (sown on May 31, 2014)				
07.07	14	18	Stem elongation	99 ± 4
11.07	13	21	Stem elongation	114 ± 4
14.07	17	23	Ear emergence	127 ± 5
25.07	16	21	Dough development	129 ± 5
Treatment (sown on July 15, 2014)				
25.07	16	21	Germination	73 ± 3
11.09	1	9	Stem elongation, ear emergence	128 ± 5
25.09	−4	1	Dough development (cold hardening phase I)	154 ± 6
30.09	−7	−3	Dough development (cold hardening phase II)	155 ± 6

*—daily air temperature.

**Table 2:** Total contents of lipids (TLs, mg/g dry weight ± standard error) in the leaves of the perennial cereal *Bromopsis inermis* growing at different temperatures, at different stages of development.

Date	t, °С *		Stages of Development	TLs, mg/g DW
	min	Average		
Control—grass without mowing				
06.06	3	12	Tillering	26 ± 2
16.06	12	16	Stem elongation	30 ± 2
11.07	13	21	Ear emergence	44 ± 2
25.07	16	21	Dough development	57 ± 3
Treatment—grass after mowing (July 15, 2014)				
25.07	16	21	Aftergrass	93 ± 3
18.08	7	16	Stem elongation	89 ± 3
11.09	1	9	Ear emergence	124 ± 5
25.09	−4	1	Dough development (cold hardening phase I)	134 ± 4
30.09	−7	−3	Dough development (cold hardening phase II)	137 ± 4

*—daily air temperature.

**Table 3:** Contents of fatty acids (mg/g of dry weight and % of total FA ± standard error) in the leaves of the perennial cereal *Bromopsis inermis* before mowing—07.07.2013 (July) and after mowing—25.09.2013 (September), and values of Student’s *t*-test (*t*).

Fatty Acids	July *	September	*t*	July	September	*t*
	mg/g	mg/g		%	%	
14:0	0.10 ± 0.06	0.14 ± 0.01	0.62	0.6 ± 0.3	0.45 ± 0.02	−0.57
16:0	3.5 ± 0.4	4.9 ± 0.3	**2.83**	20 ± 1	15.8 ± 0.3	**−2.80**
18:0	0.5 ± 0.1	0.6 ± 0.1	1.12	2.7 ± 0.3	1.8 ± 0.1	−2.56
20:0	0.19 ± 0.01	0.25 ± 0.03	1.76	1.10 ± 0.04	0.8 ± 0.1	**−3.66**
22:0	0.23 ± 0.03	0.28 ± 0.02	1.57	1.3 ± 0.1	0.91 ± 0.04	**−3.33**
16:1n-9+n-7	0.1 ± 0.1	0.27 ± 0.03	**2.91**	0.3 ± 0.3	0.9 ± 0.2	1.60
16:1n-5	0.36 ± 0.03	0.6 ± 0.1	**4.90**	2.1 ± 0.1	2.02 ± 0.01	−0.43
18:1n-9	0.6 ± 0.3	0.6 ± 0.1	−0.28	3.6 ± 1.6	1.81 ± 0.04	−1.11
18:2n-6	2.0 ± 0.2	4.1 ± 0.4	**4.87**	11.5 ± 0.4	13.0 ± 0.3	**3.12**
18:3n-3	9.7 ± 1.2	19 ± 1	**5.37**	55 ± 4	61 ± 1	1.52
SFAs	4.7 ± 0.5	6.4 ± 0.5	2.54	27 ± 2	21 ± 1	**−2.79**
MUFAs	1.2 ± 0.3	1.6 ± 0.2	1.15	6.8 ± 1.7	5.2 ± 0.2	−0.90
PUFAs	12 ± 1	23 ± 2	**5.45**	67 ± 4	74.2 ± 0.4	2.07
ΣFAs	18 ± 2	31 ± 2	**4.93**	-	-	-

* the average air temperature in July = 10.9 °С, the average air temperature in September = −0.6°С; ΣFAs—total fatty acids, SFAs—saturated fatty acids, MUFAs—monounsaturated fatty acids, PUFAs—polyunsaturated fatty acids, bold font—significant differences according to Student’s *t*-test.

**Table 4:** Content of EPA+DHA and total fatty acids (mg/100g and mg/g of wet weight, respectively) and the ratio of total omega-6 and omega-3 PUFAs in the muscle, liver, and subcutaneous adipose tissue of Yakutian horses. Means in lines labeled with the same letters are not significantly different at *p* < 0.05 after Tukey’s HSD *post hoc* test (normal distribution, standard errors are given) or Kruskal–Wallis test with multiple comparisons of mean ranks (non-normal distribution standard errors are omitted).

	Muscle	Liver	SCfat
EPA+DHA, mg/100 g ww	11 ± 1 ^A^	11 ±1 ^A^	-
Total FA, mg/g ww	31 ^A^	28 ^A^	854 ^B^
n-6/n-3	1.1 ± 0.2 ^A^	5.5 ± 1.2 ^B^	0.44 ± 0.03 ^A^

**Table 5:** Calculated desaturase and elongase activity indices.

Product/Precursor Ratio	Liver	SCfat	*t*
18:0/16:0	2.5 ± 0.3	0.19 ± 0.02	**7.48**
16:1n-7/16:0	0.20 ± 0.02	0.3 ± 0.1	**−3.85**
18:1n-9/18:0	0.42 ± 0.04	5.3 ± 0.4	**−12.81**
20:4n-6/20:3n-6	7.9 ± 0.7	-	-
20:5n-3/20:4n-3	8.8 ± 0.8	0.5 ± 0.1	**10.19**
20:5n-3/18:3n-3	0.06 ± 0.03	0.0006 ± 0.0002	**2.34**
20:4n-6/18:2n-6	0.14 ± 0.01	0.003 ± 0.001	**11.80**

Bold font—significant differences according to Student’s *t*-test.

## References

[1] Petrov K.A., Sofronova V.E., Chepalov V.A., Perk A.A., Maksimov T.K. (2010). Sezonnye izmeniniia soderzhaniia fotosinteticheskikh pigmentov u mnogoletnikh travianistykh rastenii kriolitozony [Seasonal changes in the content of photosynthetic pigments in perennial grasses of the permafrost zone]. Fiz.rast. [Russ. J. Plant Physiol.].

[2] Petrov K.A. (2016). Kriorezistentnost’ Rastenii: Ekologo-fiziologicheskie I Biokhemicheskie Aspekty [Cryoresistance of Plants: Ecological, Physiological and Biochemical Aspects].

[3] Chirkova T.V. (2002). Fiziologicheskie Osnovy Ustoichivosti Rastenii [Physiological Basis of Plant Resistance].

[4] Zakharova V.I., Kuznetsova L.V., Ivanova E.I., Vasilyeva-Kralina I.I., Gabyshev V.A., Egorova A.A., Zolotov V.I., Ivanova A.P., Ignatov M.S., Ignatova E.A. (2005). Raznoobrazie Rastitel’nogo Mira Iakutii [The Diversity of Plant Life in Yakutia].

[5] Aleksandrova V.D., Andreev V.N., Vakhtina T.V., Dydina R.A., Karev G.I., Petrovskii V.V., Shamurin V.F. (1964). Kormovaya Kharakteristika Rastenii Krainego Severa [Fodder Characteristics of the Plants of the Far North].

[6] Andreev V.N., Belyaeva N.V., Galaktionova T.F., Govorov P.M., Egorov A.D., Kurilyuk T.T., Myarikyanov M.I., Permyakova A.A., Perfilieva V.I., Petrov A.M. (1974). Tebenevochnye Pastbishcha Vostoka Iakutii [Winter-Grazing Pastures of the North-East of Yakutia].

[7] Egorov A.D., Potapov V.Y., Romanov P.A. (1962). Zonal’no-biokhimicheskie Osobennosti Kormovykh Rastenii iakutii i Nekotorye Problemy Razvitiia Zhivotnovodstva [Zonal-biochemical Characteristics of Fodder Plants in Yakutia and Some Problems in the Development of Animal Husbandry].

[8] Potapov V.Y. (1967). Uglevody i Lignin v Kormovykh Travakh Iakutii [Carbohydrates and Lignin in the Fodder Grasses of Yakutia].

[9] Pakendorf B., Novgorodov I.N., Osakovskij V.L., Danilova A.P., Protod’jakonov A.P., Stoneking M. (2006). Investigating the effects of prehistoric migrations in Siberia: Genetic variation and the origins of Yakuts. Hum. Genet..

[10] Crubézy E., Amory S., Keyser C., Bouakaze C., Bodner V., Gibert V., Röck F., Parson W., Alexeev A., Ludes B. (2010). Human evolution in Siberia: From frozen bodies to ancient DNA. BMC Evol. Biol..

[11] Keyser C., Hollard C., Gonzalez A., Fausser J., Rivals E., Alexeev A., Riberon A., Crubézy E., Ludes B. (2015). The ancient Yakuts: A population genetic enigma. Philos. Trans. R. SocLond. B Biol. Sci..

[12] Guryev I.P. (1983). K voprosu o Proiskhozhdenii Iakutskoi Loshadi. Teriologicheskie Issledovaniia v Iakutii [On the Issue of the Yakutian Horse Origin. Theriological Research in Yakutia].

[13] Knyazev S.P. (1996). Analiz geneticheskikh markerov aborigennykh iakutskikh loshadei v sviazi s filogeniei i domestikatsiei loshadei [Analysis of genetic markers of indigenous Yakutian horses in connection with the phylogeny and domestication of horses]. Proceedings of the International Conference “Animal Molecular and Genetic Markers”.

[14] Tikhonov V.N. (1998). Populiatsionno-geneticheskie parametry aborigennykh iakutskikh loshadei v sviazi s fiologeniei sovremennykh porod domashnie loshadi *Equus caballus* L. [Population and genetic parameters of indigenous Yakutian horses in connection with the physiology of modern domestic horse *Equus caballus* L.]. Genetika [Genetics].

[15] Librado P., Sarkissian C., Ermini L., Schubert M., Jоnsson H., Albrechtsen A., Fumagalli M., Yang M., Gamba C., Seguin-Orlando A. (2015). Tracking the origins of Yakutian horses and the genetic basis for their fast adaptation to subarctic environments. Proc. Natl. Acad. Sci. USA.

[16] Petrov K.A., Chepalov V.A., Sofronova V.E., Ilyin A.N., Ivanov R.V. (2017). Ekologo-fiziologicheskie i biokhimicheskie osnovy formirovaniia zelenogo kriokorma v Iakutii [Environmental, physiological and biochemical basis of green cryo-fodder formation in Yakutia]. Sel’skokhoziaistvennaia Bioloiia [Agric. Biol.].

[17] Petrov K.A., Dudareva L.V., Nokhsorov V.V., Perk A.A., Chepalov V.A., Sophronova V.E., Voinikov V.K., Zulfugarov I.S., Lee C.-H. (2016). The role of plant fatty acids in regulation of the adaptation of organisms to the cold climate in cryolithic zone of Yakutia. J. Life Sci..

[18] Alekseev N.D., Neustroev M.P., Ivanov R.V. (2006). Biologicheskie Osnovy Povysheniya Produktivnosti Loshadej [The Biological Basis for Increasing Horse Productivity].

[19] Bligh E.C., Dyer W.J. (1959). A rapid method of total lipid extraction and purification. Can. J. Biochem. Physiol..

[20] Christie W.W. (1993). Preparation of ester derivatives of fatty acids for chromatographic analysis. Adv. Lipid Methodol..

[21] Vaskovsky V.E., Kostetsky E.Y., Vasendin J.M. (1975). Universal reagent for determination of phosphorea in lipids. J. Chromatogr..

[22] Wagner H., Horhammer L., Walff P. (1961). Dunnschtchromatographic von Phosphatiden und Glykolipiden. Biochem. Z..

[23] Kates M. (1972). Techniques of Lipidology: Isolation, Analysis and Identification of Lipids.

[24] Makhutova O.N., Borisova E.V., Shulepina S.P., Kolmakova A.A., Sushchik N.N. (2017). Fatty acid composition and content in chironomid species at various life stages dominating in a saline Siberian lake. Contemp. Probl. Ecol..

[25] Adolph S., Schedlbauer C., Blaue D., Schöniger A., Gittel C., Brehm W., Fuhrmann H., Vervuert I. (2019). Lipid classes in adipose tissues and liver differ between Shetland ponies and Warmblood horses. PLoS ONE.

[26] Murata N., Los D.A. (1997). Membrane fluidity and temperature perception. Plant Physiol..

[27] Tocher D.R., Leaver M.J., Hodson P.A. (1998). Recent advances in the biochemistry and molecular biology of fatty acyl desaturase. Prog. Lipid Res..

[28] Murata N., Los D.A. (2006). Genome-wide analysis of gene expression characterizes histidine kinase Hik33 as an important component of the cold-signal transduction in cyanobacteria. Physiol. Plant.

[29] Guschina I.A., Harwood J.L., Arts M.T., Kainz M., Brett M.T. (2009). Algal lipids and effect of the environment on their biochemistry. Lipids in Aquatic Ecosystems.

[30] Trunova T.I. (2007). Rastenie i Nizkotemperaturnyi Stress [The Plant and the Stress Caused by Low Temperatures].

[31] Sofronova V.E., Chepalov V.A., Petrov K.A., Dymova O.V., Golovko T.K. (2019). Fond zelenykh i zheltykh pigmentov iarovogo ovsa, kul’tiviruemogo dlia polucheniia kriokorma v usloviiakh Tsentral’noi Iakutii [Green and yellow pigments of spring oats cultivated as cryo-fodder in the conditions of Central Yakutia]. Agrarny Vestnik Urala [Ural Agric. Bull.].

[32] Petrov K.A., Perk A.A., Chepalov V.A., Chapter I.V., Onakpoya I. (2012). Linoleic and Other Fatty Acids, Cryoresistance, and Fodder Value of Yakutian Plants. Linoleic Acids. Sources, Biochemical Properties and Health Effects.

[33] Dudareva L.V., Rudikovskaya E.G., Nokhsorov V.V., Petrov K.A. (2015). Fatty- acid profiles of aerial parts of three horsetail species growing in Central and Northern Yakutia. Chem. Nat. Compd..

[34] Nokhsorov V.V. (2017). Adaptivnye izmeneniia sostava i soderzhaniia lipidov rastenii kriolitozony Iakutii pri gipotermii [Adaptive changes in the composition and content of lipids in the plants of the cryolithic zone in Yakutia in the hypothermal conditions]. Ph.D. Thesis.

[35] Welti R., Li W., Li M., Sang Y., Biesiada H., Zhou H.-E., Rajashekar C.B., Williams T.D., Wang X. (2002). Profiling membrane lipids in plant stress responses—Role of phospholipase Dα in freezing-induced lipid changes in Arabidopsis. J. Biol. Chem..

[36] Wang S.Y., Lin H.-S. (2006). Effect of plant growth temperature on membrane lipids in strawberry (Fragaria × ananassa Duch.). Sci. Hortic..

[37] Vereshchagin A.G. (2007). Lipidy v Zhizni Rastenii [Lipids in Plant Life].

[38] Gabyshev M.F. (1957). The Yakut Horse.

[39] Aleekseev N.D. (2005). Novoe o proiskhozhdenii loshadei iakutskoi porody (biologicheskie aspekty [New in the origin of the Yakutian horses (biological aspects)]. Nauka i Obrazovanie [Sci. Educ.].

[40] Gladyshev M.I., Sushchik N.N., Makhutova O.N. (2013). Production of EPA and DHA in aquatic ecosystems and their transfer to the land. Prostaglandins Other Lipid Mediat..

[41] Twining C.W., Brenna J.T., Hairston N.G., Flecker A.S. (2016). Highly unsaturated fatty acids in nature: What we know and what we need to learn. Oikos.

[42] Malcicka M., Visser B., Ellers J. (2018). An evolutionary perspective on linoleic acid synthesis in animals. Evol. Biol..

[43] Tocher D.R., Dick J.R., MacGlaughlin P., Bell J.G. (2006). Effect of diets enriched in Δ6 desaturated fatty acids (18:3n-6 and18:4n-3), on growth, fatty acid composition and highly unsaturated fatty acid synthesis in two populations of Arctic charr (*Salvelinus alpines* L.). Comp. Biochem. Physiol. Part B.

[44] Suagee J.K., Corl B.A., Crisman M.V., Wearn J.G., McCutcheon L.J., Geor R.J. (2010). De novo fatty acid synthesis and NADPH generation in equine adipose and liver tissue. Comp. Biochem. Physiol..

[45] Mordovskaya V.I., Krivoshapkin V.G., Pogozheva A.V., Baiko V.G. (2005). Fatty acid composition of adipose tissue lipids in horses of Yakut breed. Vopr. Pitan..

[46] Slobodchikova M.N., Ivanov R.V., Stepanov K.M., Pustovoy V.F., Osipov V.G., Mironov S.M. (2011). Lipid fatty acid composition of the fat tissue of the Yakut horse. HorseBreed. Eques. Sport..

[47] Ghandour R.A., Colson C., Giroud M., Maurer S., Rekima S., Ailhaud G., Klingenspor M., Amri E.-Z., Pisani D.F. (2018). Impact of dietary ω3 polyunsaturated fatty acid supplementation on brown and brite adipocyte function. J. Lipid Res..

[48] Colson C., Ghandour R.A., Dufies O., Rekima S., Loubat A., Munro P., Boyer L., Pisani D.F. (2019). Diet supplementation in ω3 polyunsaturated fatty acid favors an anti-inflammatory basal environment in mouse adipose tissue. Nutrients.

[49] Pisani D.F., Ailhaud G. (2019). Involvement of polyunsaturated fatty acids in the control of energy storage and expenditure. Oilseeds Fats Crop. Lipids.

[50] Belaunzaran X., Lavín P., Mantecón A.R., Kramer J.K.G., Aldai N. (2018). Effect of slaughter age and feeding system on the neutral and polar lipid composition of horse meat. Animal.

[51] Tonial I.B., Aguiar A.C., Oliveira C.C., Bonnafé E.G., Visentainer J.V., de Souza N.E. (2009). Fatty acid and cholesterol content, chemical composition and sensory evaluation of horsemeat. S. Afr. J. Anim. Sci..

[52] Lorenzo J.M., Sarriés M.V., Tateo A., Polidori P., Franco D., Lanza M. (2014). Carcass characteristics, meat quality and nutritional value of horsemeat: A review. Meat Sci..

[53] Belaunzaran X., Bessa R.J.B., Lavín P., Mantecón A.R., Kramer J.K.G., Aldai N. (2015). Horse-meat for human consumption—Current research and future opportunities. Meat Sci..

[54] Ferjak E.N., Cavinder C.A., Sukumaran A.T., Burnett D.D., Lemley C.O., Dinh T.T.N. (2019). Fatty acid composition of mesenteric, cardiac, abdominal, intermuscular, and subcutaneous adipose tissues from horses of three body condition scores. Livest. Sci..

[55] De Caro J., Eydoux C., Chérif S., Lebrun R., Gargouri Y., Carrière F., De Caro A. (2008). Occurrence of pancreatic lipase-related protein-2 in various species and its relationship with herbivore diet. Comp. Biochem. Physiol. Part B.

[56] Juárez M., Polvillo O., Gómez M.D., Alcalde M.J., Romero F., Valera M. (2009). Breed effect on carcass and meat quality of foals slaughtered at 24 months of age. Meat Sci..

[57] Guil-Guerrero J.L., Rincón-Cervera M.A., Venegas-Venegas C.E., Ramos-Bueno R.P., Suárez M.D. (2013). Highly bioavailable α-linolenic acid from the subcutaneous fat of the Palaeolithic Relict “Galician horse”. Int. Food Res. J..

[58] Belaunzaran X., Lavín P., Barron L.J.R., Mantecon A.R., Kramer J.K.G., Aldai N. (2017). An assessment of the fatty acid composition of horse-meat available at the retail level in northern Spain. Meat Sci..

[59] Gladyshev M.I., Makhutova O.N., Gubanenko G.A., Rechkina E.A., Kalachova G.S., Sushchik N.N. (2015). Livers of terrestrial production animals as a source of longchain polyunsaturated fatty acids for humans: An alternative to fish?. Eur. J. Lipid Sci. Technol..

[60] Del Bò C., Simonetti P., Gardana C., Riso P., Lucchini G., Ciappellano S. (2013). Horse meat consumption affects iron status, lipid profile and fatty acid composition of red blood cells in healthy volunteers. Int. J. Food Sci. Nutr..

[61] Lee C.-E., Seong P.-N., Oh W.-Y., Ko M.-S., Kim K.-I., Jeong J.-H. (2007). Nutritional characteristics of horsemeat in comparison with those of beef and pork. Nutr. Res. Pract..

[62] Lorenzo J.M., Munekata P.E.S., Campagnol P.C.B., Zhu Z., Alpas H., Barba F.J., Tomasevic I. (2017). Technological aspects of horse meat products—A review. Food Res. Int..

